# Memory capacity as the core mechanism of the development of space–time interferences in children

**DOI:** 10.1038/s41598-024-61018-1

**Published:** 2024-05-06

**Authors:** Quentin Hallez, Fuat Balcı

**Affiliations:** 1 Laboratoire Développement, Individu, Processus, Handicap, Éducation (DIPHE), Université Lumière Lyon 2, 5 Avenue Pierre Mendès France, 69500 Bron, France; 2 Department of Biological Sciences, University of Manitoba, Winnipeg, MB Canada; 3 Department of Psychology, Koç University, Istanbul, Turkey

**Keywords:** Development, Cognition, Perceptions, Space–time, Short-term memory, Children, Neuroscience, Psychology

## Abstract

This study investigated the development of spatiotemporal perceptual interactions in 5-to-7 years old children. Participants reproduced the temporal and spatial interval between sequentially presented visual stimuli. The time and spacing between stimuli were experimentally manipulated. In addition, cognitive capacities were assessed using neuropsychological tests. Results revealed that starting at 5 years old, children exhibited spatial biases in their time estimations and temporal biases in their spatial estimations, pointing at space–time interference. In line with developmental improvement of temporal and spatial abilities, these spatiotemporal biases decreased with age. Importantly, short-term memory capacity was a predictor of space–time interference pointing to shared cognitive mechanisms between time and space processing. Our results support the symmetrical hypothesis that proposes a common neurocognitive mechanism for processing time and space.

## Introduction

Space and time are integral dimensions of perceptual experience and thereby shape our interactions with the world. Resultantly veridical representations of time and space (e.g., distance) are critical for adaptive planning and execution of goal directed actions. Although space and time are considered as orthogonal dimensions (but see the relativity theory), they are not necessarily processed independently in the brain. For instance, spatial features of events can bias the subjective estimates of their durations, and vice versa. The mechanisms underlying these interrelations are far from being well understood. The current study aims to provide a developmental insight into the cognitive mechanism that underlies the spatiotemporal interactions. To this end, active acquisition and refining of perceptual and cognitive abilities during cognitive development offer a unique window for understanding the emergence and modulation of spatiotemporal interferences.

The contemporary debate concerning the interactions between time and space are shaped around two opposing hypotheses: the asymmetric *vs.* symmetric interaction hypothesis. According to the asymmetric interaction hypothesis, rooted in the Conceptual Metaphor Theory (CMT) of Lakoff and Johnson^1^, individuals utilize spatial metaphors to conceptualize, think about, and communicate time, leveraging their concrete spatial experiences to support the understanding of time as an abstract quantity^2^. For instance, expressions such as “This is ahead of its time” or “This event is now behind us” involves the use of spatial metaphors to structure temporal concepts. Crucially, the “asymmetric” interaction hypothesis does not lend support for the use of time to describe spatial relations given the abstract nature of time. This hypothesis has received widespread support based on numerous behavioral studies demonstrating the influence of space on time while finding no evidence of time affecting spatial perception^3^. Accordingly, the *asymmetric interaction* hypothesis suggests that time and space are processed by distinct cognitive mechanisms or temporal representations are embedded in spatial information processing^4^.

On the other hand, the *symmetrical* interaction hypothesis proposes that spatial and temporal perceptions can mutually interfere with each other leading to bidirectional biasing effects. This idea was first alluded to by “A Theory of Magnitude” (ATOM)^5^, which offers a unified sense of magnitude underlying reasoning about space, time, and numbers. The primary mechanistic support for this hypothesis comes from the field of cognitive neuroscience. For instance, functional neuroimaging studies have shown similar activations of the parietal cortex during the processing time, space, and numbers^6–8^. Other studies showed mapping between numerical magnitude and spatial length in as young as 8-month-old infants^9^. This view is further supported by similar degrees of discriminability observed for time, numerosity, and size (e.g., 2:1 ratio in 6-month-old and 3:2 ratio in 9-month-old infants^10–13^) and cross-model mappings between time, numerosity and length in 8- and 9-month old infants^14,15^ (also see^16^ for a review). However, specificities in the development of number sense challenge this hypothesis^17,18^. Several researchers concluded that numbers (as discrete quantities) exhibit distinct characteristics compared to the continuous dimensions of time and space, although this assumption has not been systematically verified. Since then, only a few behavioral studies have provided evidence in favor of the *symmetric interaction hypothesis*^19^.

The demonstration of interdependencies between the perception of spatial and temporal features dates to several decades ago. In early studies, three stimuli were presented sequentially using three light bulbs on a wall^20–22^. The sequential light flashing between the three bulbs established two space–time intervals between neighboring light bulbs. Researchers showed that the distance between two light bulbs was perceived longer if it was accompanied with a longer temporal interval (known as the *tau* effect)^23^. Reciprocally, researchers also demonstrated that the temporal interval between two stimuli is perceived as longer if it is accompanied with a longer distance (known as the *kappa* effect).

The foundational work of Casasanto and Boroditsky^3^ provides compelling evidence for the *asymmetrical* hypothesis in the context of these well-established effects. Specifically, they showed that although *kappa* and *tau* effect can co-occur, the latter is often associated with larger effects. Similar results were reported in children^24,25^. In these studies, children were asked to observe videos of two snails moving along parallel paths of varying distances and durations. After each video, the children were asked to determine which snail had traveled for a longer time or a longer distance. Corroborating the findings gathered from adults, the results of these studies indicated that the perception of time was more strongly influenced by distances.

One potential source of superiority of space over time is different cognitive demands exerted by spatial *vs.* temporal tasks. Although researchers attempted to control the discriminability of stimuli and the complexity of responses in two domains, temporal tasks might require more cognitive resources than spatial tasks (e.g., due to its abstract nature), leading to more effortful judgments for similar levels of accuracy. For instance, considering the presumed dependance of time and space on a shared neurocognitive substrate, one cannot ascertain the equivalence of the resultant perceptual uncertainty associated with temporal and spatial contexts. For example, the subjective discriminability of 1–4 s might differ from the subjective discriminability of 100 and 400 pixels.

The current experiment aimed to test this hypothesis by analyzing, within each dimension, if similar cognitive capacities can predict the interference caused by the concurrent dimension. Demonstrating the moderation of the effects by a common cognitive component would provide an alternative explanation to the prediction of the *asymmetrical* hypothesis by implicating the involvement of common processes for temporal and spatial information processing^26^. Additionally, the outcomes of this critical test would provide valuable insights regarding the locality of the spatiotemporal interference. To this end, two primary sources of interference has been assumed regarding space–time interaction. These sources correspond to different stages of most magnitude perception models (i.e., the pacemaker-accumulator models^27^).

The first stage of interference can occur in an online fashion during the encoding of magnitudes within a neural format^28^. If the same mechanism underlies the processing of time and space, then a greater amount of attention should be needed to treat both dimensions without reducing accuracy^29–33^. To this end, recent developmental studies showed that the underestimation of time caused by reduced attention was directly related to children’s individual attentional capacities^34,35^. Based on this rationale, participants with lower attention scores are expected to be more susceptible to space–time interference. Alternatively, space–time interference may also occur at a later stage during which temporal and spatial information are maintained in short-term memory^36–41^ without ruling out the independent effect of attention.

In the current study, 5- to 7-year-old children reproduced both time and distances established by the sequential presentation of three stimuli. The time interval and the distance between the stimuli were systematically manipulated. This age range was chosen for several reasons. Firstly, development over the course of this age range exhibit significant heterogeneity in terms of cognitive capacity, allowing for a more comprehensive examination of the influence of individual cognitive abilities. Secondly, explicit time perception reaches a level of maturity during this age range, making it feasible to conduct tasks involving time reproductions^42,43^.

We hypothesized that (1) increasing the distance between stimuli will lead to the overestimation of durations, and (2) this effect would be more pronounced in younger children due to less developed cognitive capacity. Conversely, we expected (3) an overestimation of distance between the sequence of stimuli when it was accompanied by longer time interval, and (4) that this effect would be more pronounced in younger children due to less developed cognitive capacity. Based on the assumption of a common encoding mechanism, we expected (5) the biasing effect of time on distance estimations or the biasing effect of distance on time estimations will interact with individual attention scores. Finally, if spatiotemporal interference occurs at the memory stage, (6) these bidirectional biasing effects are expected to interact with memory measures.

## Method

### Participants

A total of 82 children were recruited. Twenty-two children were 5 years olds (*M* = 5.64, *SD* = 0.24, 11 females), 29 were 6 years olds (*M* = 6.43, *SD* = 0.31, 12 females) and 31 were 7 years olds (*M* = 7.56, *SD* = 0.38, 16 females). The sample size was determined based on an a priori power analysis conducted using G*power software^35^ for a main experimental design with within-between subject interactions. The analysis indicated that a total sample size of 69 participants was required, considering the effect size (η^2^p = 0.03), desired power (0.95), number of groups (3), number of measurements (6), and the correlation among repeated measures (0.5). Since previous studies did not find an age effect, the choice of the smallest effect size for the power analysis was guided by theoretical considerations based on a thorough review of the existing literature and relevant theoretical frameworks^34,35, 44, 45^. Thus, this effect size was chosen based on its theoretical significance and practical relevance in investigating such an effect. The young children were recruited from a nursery school and the older children were recruited from a primary school in […] (this part was hidden to respect authors' anonymity). The children’s parents signed written informed consent for the participation of their dependents in this study. The study was carried out according to the principles of the Helsinki Declaration and was approved by both the Department of National Education Services and the research ethics committee of the University […] (this part was hidden to respect authors' anonymity).

## Material

The experiment employed a 33 cm computer with a resolution of 1920 × 1080 to control the presentation of stimuli and to record responses using a compiled Python program stored on the hard drive. Participants were tested in a quiet room adjacent to the classroom. The stimuli consisted of a series of three white circles (4 cm in diameter), appearing rhythmically with a fixed delay of 700, 1100, or 1500 ms, and with spacing of 200, 300, or 400 pixels. Responses for both temporal and spatial estimations were collected using a computer mouse. The short-term and working memory performance was assessed with the computerized subtests of the Corsi Block-Tapping Test^46^ programmed in Python by the first author. The attention performance was assessed using a sub-test of the TEA-Ch (Test of Everyday Attention for Children) known as “sky search”^47^.

### Procedure

The children were tested over two consecutive days. They were individually invited to the study during their classroom activities. On the first day, the order of time and space estimation tasks was pseudo-randomized with the constraint of counterbalancing the assignment across participants. Subsequently, the children completed the neuropsychological tests described in the section below. On the second day, the children were again invited individually during the same time slot. Children completed the temporo-spatial task by estimating the dimension they had not performed on the first day of testing.

### Temporo-spatial task

**Figure 1:**
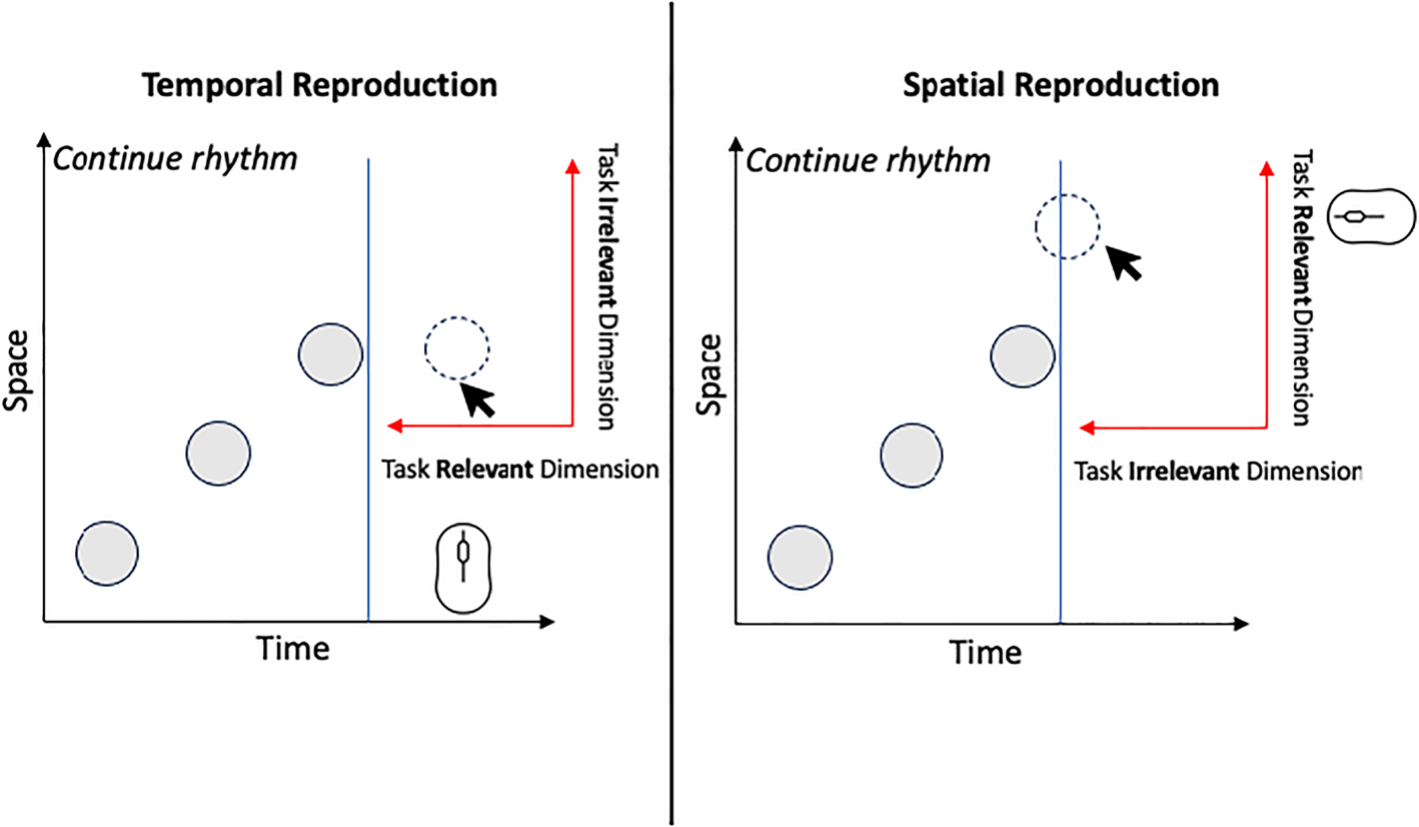
Illustration of the experimental setup for the temporal reproduction task (left) and spatial reproduction task (right).

In both temporal and spatial estimation task (cf Fig. 1), three white circles were displayed on a black background successively according to experimentally determined time and spatial intervals between each two successive presentations. The time and spatial intervals stayed constant within a trial.

During the temporal estimation session, children were instructed to click on a computer mouse to maintain the rhythm generated by the programmed sequential presentation of the three white circles. In the spatial estimation session, children were asked to continue the spatial pattern displayed by the three white circles. To accomplish the spatial estimation, a cursor appeared after the exposure of the third circle, allowing participants to aim and click on the desired location. At the beginning of each session, children performed a total of six training trials, including three demonstration trials conducted by the experimenter and three learning trials performed by the participant with the assistance of the experimenter. During testing, each experimental condition was presented five times resulting in a total of 45 trials for each session (3 durations × 3 spaces × 5 repetitions = 45 trials).

### Corsi block-tapping test

Children were presented with a grid of nine squares, which sequentially lit up one by one to form a specific sequence. Participants were asked to immediately reproduce the sequence by clicking on corresponding squares in both forward (short-term memory) and backward order (working memory). The test comprised a total of eight block-tapping sequences, ranging from two to nine blocks, with two trials per sequence. The test was concluded when the participant failed to reproduce the correct sequence on two consecutive trials of the same sequence. The scores associated with each of the sub-test corresponds to the total of correct answers (*i.e.,* 1 point for each correct answer).

### Sky search

Sky search task was used to assess visual selective attention^47^. Participants were instructed to encircle all pairs of identical spaceships on a board comprising a set of 130 pairs (comprising 20 targets and 110 lures) as fast as possible. This board was used in conjunction with another board that exclusively featured 20 pairs of target spaceships. The performance was calculated as follows: (completion time in seconds to complete the board with lures/spaceships found on this specific board)–(completion time for target-only board/spaceships found on this specific board). The purpose of this calculation is to extract the completion time associated with the motor component from the overall score.

### Ethics approval

The study was carried out according to the principles of the Helsinki Declaration and was approved by both the Department of National Education Services and the research ethics committee of Comité CER-UdL n° 2023-04-06-006 [blinded for the peer review process].

## Results

All research data are publicly available via an anonymous Open Science Framework (OSF) link. The link can be accessed by visiting [https://osf.io/neyt6/?view_only=ddf6ecfc4df44ce980663c4394cbc2f4]. Data were analyzed using JASP v 0.17.1.^48^ The measure associated with the temporal estimation task corresponds to the participant's temporal error, which is calculated as: participant's Estimation—Presented Time.

To identify potential outliers, boxplots were generated for each condition and age group. Outliers, according to the boxplot method, are defined as data points lying outside the range [Quartile 1 − 1.5 * (Interquartile Range); Quartile 3 + 1.5 * (Interquartile Range)]. A total of 178 productions were excluded (4.82%) using this method. Specifically, 58 productions of 5-year-olds (out of 990, 5.86%), 65 productions of 6-year-olds (out of 1305, 4.98%), and 55 productions of 7-year-olds were excluded from the analyses (out of 1395, 3.94%). Overall, all outlier data correspond to over-productions. No under-produced data was detected (the shortest duration produced is 621 ms, which does not appear aberrant given the employed method).

As for the spatial task, the employed measure corresponded to the number of pixels between the participant's estimation and the fourth circle, divided by the number of pixels on the screen (to normalize by the screen size), all on the horizontal axis. A total of 134 productions were rejected (3.63%), with 45 rejections (80% underestimated values vs 20% overestimated) in the 5-year-olds group (4.55%), 60 rejections (60% underestimated values vs. 40% overestimated) in the 6-year-olds group (4.6%), and 29 rejections (34.5% underestimated values vs. 65.5% overestimated) in the 7-year-olds group (2.08%). No participant was excluded from the analyses (except for the last section, where two participants were excluded, as further described). Bidirectional paired t-tests on temporal reproductions suggested a Vierordt effect, where smaller distances consistently elicited overestimations compared to larger distances (200 px vs. 300 px: *t*(81) = 14.19, *p* < 0.001; 300 px vs. 400 px: *t*(81) = 11.83, *p* < 0.001).

We performed a mixed design ANOVA, incorporating the between-subject variable of sequence presentation order (children who commenced with spatial estimations versus those who began with temporal estimations), alongside the within-subject variables of time (700, 1100, and 1500 ms) and space (200, 300, and 400 pixels). This was done to analyze potential effect order. The ANOVA on spatial error shows no effect of session order (*ps* > 0.10). However, the ANOVA on time estimations errors reveal a main effect of sequence order *F*(1, 80) = 7.35, *p* = 0.008, *η*^2^ = 0.05. Indeed, children who start with the temporal task generate shorter estimations error (*M* = 832 ms) compared with children who started with the spatial task (*M* = 1164). Nonetheless, besides the disparities in temporal estimation, the order did not significantly affect the temporal error in either spatial or temporal dimensions (*ps* > 0.10), thereby reducing its impact on the subsequent results. Indeed, incorporating the within-subject variable "session order" into the subsequent ANOVA did not modify the reported findings.

### Spatial influences on temporal reproductions

**Figure 2:**
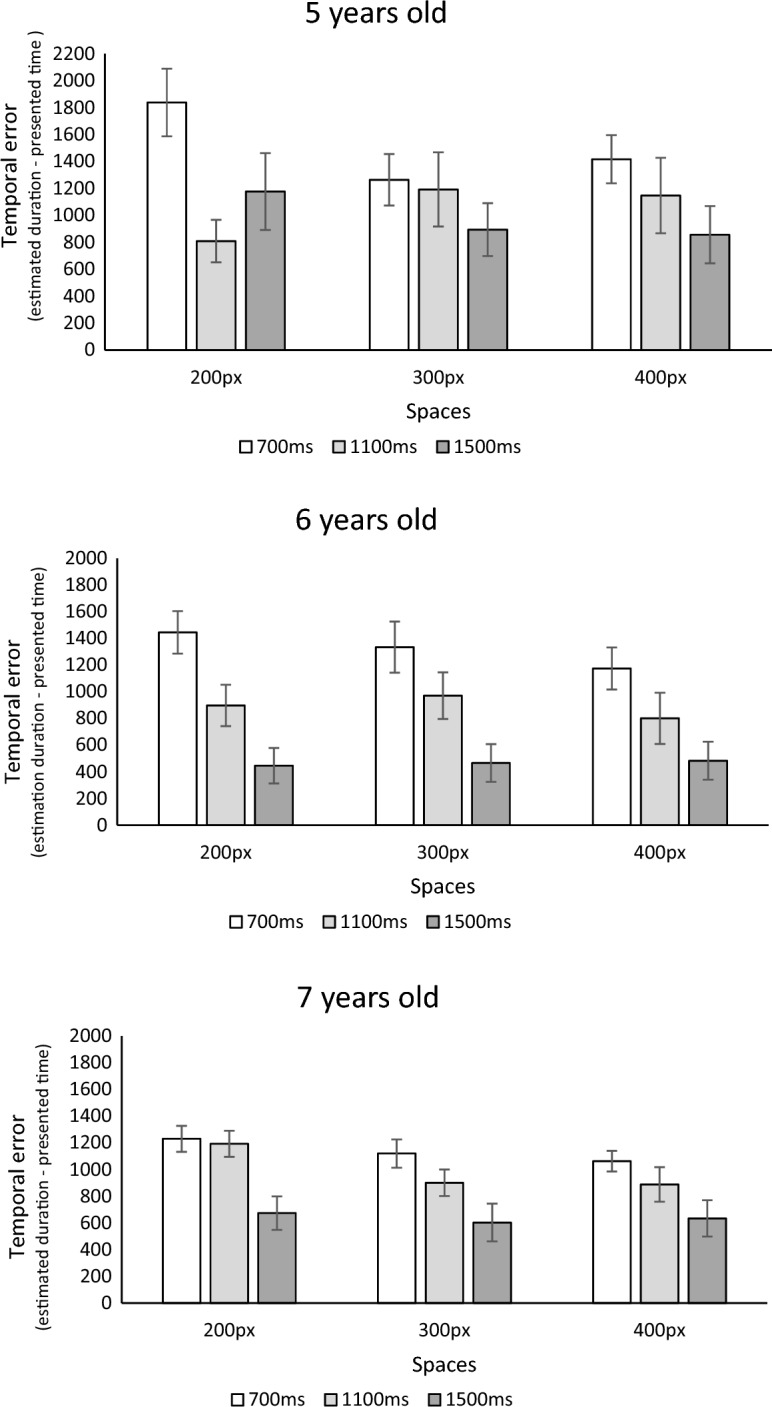
Illustration of the temporal error associated with the three duration conditions (700, 1100, and 1500 ms) along with the spatial conditions (200, 300, 400 pixels) for children aged 5 years (top panel), 6 years (middle panel), and 7 years (bottom panel). Error bars represent the standard deviation.

An ANOVA was performed on time estimation errors with two within-subject variables, namely durations (700, 1100, 1500 ms) and distance (200, 300, or 400 pixels), along with the between-subject factor of age (5, 6, and 7 years). As illustrated Fig. 2, there was a main effect of the duration *F*(2, 81) = 127.59, *p* < 0.001; *η*^2^ = 0.12; *η*^2^_*p*_ = 0.62 and a significant duration × age interaction *F*(4, 81) = 4.46, *p* = 0.002; *η*^2^ = 0.01; *η*^2^_*p*_ = 0.10. Bidirectional paired t-tests showed that the overestimation of time decreased with longer durations; there were higher overestimations for the 700 ms duration (*M* = 1300.22; *SD* = 62.70) than for the 1100 ms duration (*M* = 967.15, *SD* = 648.25) *t*(81) = 8.52, *p* < 0.001, which was higher than the overestimation observed for the 1500 ms duration (*M* = 667.13, *SD* = 612.37) *t*(81) = 8.20, *p* < 0.001. The 6 year olds were more prone to a reduction of the overestimation as a function of target durations as indicated by bidirectional independent sample t-tests (*ps* < 0.05). The temporal error rate between durations were not statistically different between 5- and 7-year-olds (*p* > 0.10).

Of particular interest, the ANOVA revealed a significant effect of space *F*(2, 81) = 11.92, *p* < 0.001; *η*^2^ = 0.01; *η*^2^_*p*_ = 0.13. Contrary to our expectations, durations were underreproduced with increasing distance between stimuli (Fig. 1). Specifically, stimuli presented with a spacing of 200 pixels resulted in a larger overestimation of time (*M* = 1056; *SD* = 600.69) compared to the distances of 300 (*M* = 956.91; *SD* = 600.71) and 400 pixels (*M* = 921.58, *SD* = 594.25), as revealed by the bidirectional paired t-tests;* t*(82) = 2.70, *p* = 0.008 and *t*(82) = 3.93, *p* < 0.001, respectively. The difference between the 300 and 400 pixel conditions was not significant, *t*(82) = 1.23, *p* = 0.22.

The effect of distance interacted with age, *F*(4, 81) = 5.09, *p* < 0.001; *η*^2^ = 0.01; *η*^2^_*p*_ = 0.11. Specifically, when calculating the distortion index between the distances (i.e., 200–400 pixels reproductions; other calculation methods including all distances yielding the same result), bidirectional independent t-tests suggested that 5-year-old children were more susceptible to the effect of distance on time reproductions (*Mdiff* = 293.92, *SD* = 269.09) compared to 6 (*Mdiff* = 80.33, *SD* = 335.30) and 7-year-olds (*Mdiff* = 80.33, *SD* = 335.30) (with *t*(49) = 2.45, *p* = 0.018 and *t*(51) = 2.90, *p* = 0.005, respectively). There was no significant difference between 6 and 7-year-olds, *t*(58) = 0.11, *p* = 0.91.

Similarly, the ANOVA revealed an age-dependent trend, *F*(4, 81) = 5.09, *p* < 0.052; *η*^2^ = 0.01; *η*^2^_*p*_ = 0.1. Specifically, 5-year-old children had a higher level of time estimation error throughout the experiment (*M* = 1230.64, *SD* = 776.75) compared to 7-year-olds (*M* = 889.85, *SD* = 364.90), *t*(51) = 2.14, *p* = 0.037. The 5-year-old children also tended to make more errors in time estimations than 6-year-olds (*M* = 881.04, *SD* = 533.73), *t*(49) = 1.90, *p* = 0.06. There was no significant difference between 6 and 7-year-olds, *t*(58) = − 0.075, *p* = 0.94. These results corroborate the results of earlier developmental studies and suggest increased temporal precision with age. Finally, ANOVA indicated a significant duration × space interaction effect, *F*(4, 81) = 3.09, *p* = 0.02; *η*^2^ = 0.005; *η*^2^_*p*_ = 0.04, and a non-significant effect of the three-way interaction *F*(8, 81) = 0.63, *p* = 0.75.

### The modulation of the effect of space on time perception by cognitive components

The primary units of analysis was temporal drift caused by distances, resulting in the squared difference of distance changes: (Time Reproduction_200 px_ − Time Reproduction_300 px_)^2^ + (Time Reproduction_300 px_ − Time Reproduction_400 px_)^2^. This measure has the advantage of explaining a decrease in precision caused by distances in statistical models. If we had only considered the difference between distances or included the cognitive variable in an ANCOVA, our focus would have been on the perspective of distance on time bias measure going from underestimation to overestimation. For our theoretical perspective, it is not relevant and more valuable to preserve the precision criterion at zero in order to investigate whether a cognitive variable decreased the interference or not.

The first regression analysis of the effect of attention on the space-on-time interference was not significant *F*(1, 75) = 1.95, *p* = 0.17. Nonetheless, the overall model with short-term memory was statistically significant *F*(2, 81) = 12.78, *p* < 0.001, *η*^2^ = 0.14. Short-term memory score was a significant predictor of the space–time interference (*β* = − 0.37, *p* < 0.001), indicating the reduction of interference effects with higher short-term memory capacity. Interestingly, although less prominent (F(1, 81) = 5.62, *p* = 0.02, *η*^2^ = 0.07) similar effects were found with working memory score (*β* = −  0.26, *p* = 0.020).

### Temporal influences on spatial reproductions

An ANOVA was conducted on children’s distance reproductions, considering the within-subject factor of spacing (200, 300, or 400 pixels) and the within-subject factor of time (700, 1100, or 1500 ms). The age group was included as a between-subject factor (5, 6 and 7 years old).

**Figure 3:**
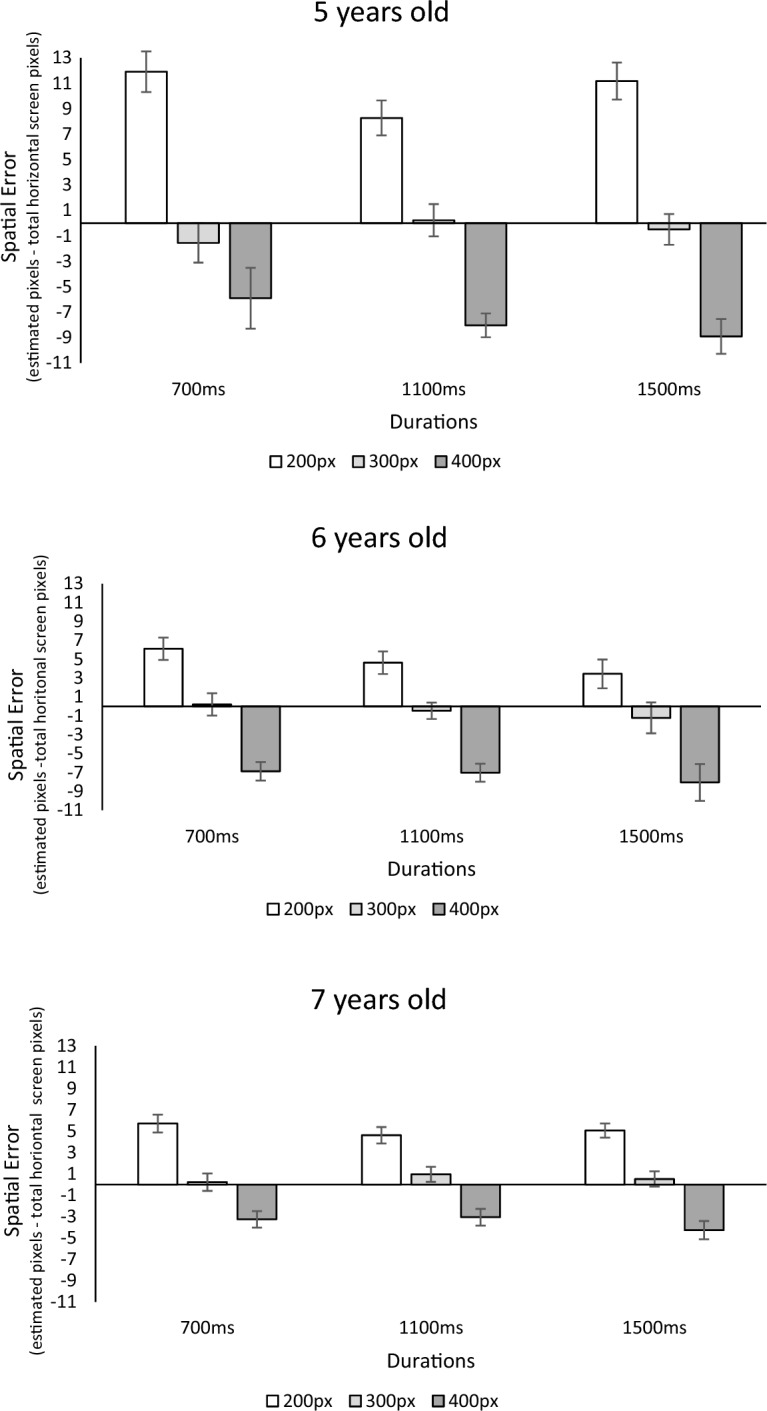
Illustration of the spatial error associated with the three duration conditions (700, 1100, and 1500 ms) along with the spatial conditions (200, 300, 400 pixels) for children aged 5 years (top panel), 6 years (middle panel), and 7 years (bottom panel). Error bars represent the standard deviation.

As illustrated Fig. 3, the model revealed a significant main effect of space *F*(2, 81) = 323.33, *p* < 0.001; *η*^2^ = 0.53; *η*^2^_*p*_ = 0.81. Furthermore, the effect of distance interacted with age *F*(4, 81) = 14.87, *p* < 0.001; *η*^2^ = 0.05; *η*^2^_*p*_ = 0.28), suggesting that the Vierordt effect was modulated with age. Independent t-tests on the difference index of distance (i.e., 200–400 px estimation) revealed a progressive decrease in the Vierordt effect with age. Similar results were found using other difference indices. Specifically, there was a more pronounced Vierordt effect in 5-year-olds (*M* = 18.06, *SD* = 7.84) compared to 6-year-olds (*M* = 12.05, *SD* = 6.34), *t*(49) = 3.03, *p* = 0.004, which was in turn stronger compared to 7-year-olds (*M* = 8.66, *SD* = 3.99), *t*(58) = 2.50, *p* = 0.01. Additionally, the ANOVA showed a significant main effect of age *F*(2, 81) = 4.62, *p* = 0.01; *η*^2^ = 0.014; *η*^2^_*p*_ = 0.11, indicating that the younger age group consistently generated greater errors compared to their older peers (bidirectional independent t-tests, *ps* < 0.05).

There was also a significant main effect of duration on distance reproductions *F*(2, 81) = 11.45, *p* < 0.001; *η*^2^ = 0.01; *η*^2^_*p*_ = 0.13). Bidirectional paired t-tests showed that the shorter duration of 700 ms resulted in overestimation of distance (*M* = 0.84, *SD* = 3.51) compared to both 1100 ms (*M* = − 0.04, SD = 3.25; *t*(81) = 3.07, *p* = 0.003) and 1500 ms (*M* = − 0.37, *SD* = 3.58; *t*(81) = 4.31, *p* < 0.001). However, the difference between 1100 and 1500 ms was not significant *t*(81) = 1.06,* p* = 0.29. Interestingly, this effect interacted with age, *F*(4, 81) = 4.33, *p* = 0.002; *η*^2^ = 0.005; *η*^2^_*p*_ = 0.10). Additional bidirectional independent t-tests were conducted on the index of distance drift caused by durations (i.e., distance reproduction_700 ms_—distance reproduction_1500ms_). The t-tests revealed that the effect of distance on time reproductions decreased with age, with 5-year-olds being more impacted by time (*M* = − 2.30, *SD* = 3.20) compared to 7 years old (*M* = − 0.45, *SD* = 1.59) *t*(51) =  − 2.77, *p* = 0.008. The developmental effect appeared to be progressive since the differences were not significant when comparing 5-year-olds with 6-year-olds (*t*(49) =  − 1.40, *p* = 0.17), as well as when comparing 6-year-olds with 7-year-olds (*t*(58) =  − 1.32, *p* = 0.019). Finally, the ANOVA did not reveal significant Distance × Time interaction effect *F*(4, 81) = 2.18, p = 0.07, nor Distance × Time × Age three way interaction *F*(8, 81) = 1.27, *p* = 0.26.

### The modulation of effect of time on space perception by cognitive components

Table 1 shows the mean score obtained by the participants on the different neuropsychological tests used for each age group.

**Table 1:** Mean, standard deviation, minimum and maximum of raw scores of different neuropsychological tests.

6 years			5 years			7 years		
Mean	S.D	[ Min;Max]	Mean	S.D	[ Min;Max]	Mean	S.D	[ Min;Max]
Sky search								
7.35	2.54	[2.25;13.42]	6.67	2.30	[3.08;11.95]	5.99	2.58	[3.00;11.76]
Short term memory								
2.41	1.87	[0;6]	4.07	2.15	[0;8]	5.84	1.61	[3;9]
Working memory								
1.95	2.42	[0;8]	3.41	1.88	[0;6]	4.42	2.61	[1;9]

The correlation analyses demonstrate that age (in months) predicts increases in all neuropsychological scores: attention (*r* = − 0.24, *p* = 0.04), short-term memory (*r* = 0.59, *p* < 0.001), or working memory (*r* = 0.42, *p* < 0.001). Note here that the correlation is negative for attention, as a lower numerical score indicates better attentional abilities.

Following the same logic as in the previous section, we calculated the spatial drift caused by temporal factors, resulting in the squared difference of space changes; (Distance Reproduction_700 ms_ − Distance Reproduction_1100 ms_)^2^ + (Distance Reproduction_1100 ms_ − Distance Reproduction_1500 ms_)^2^. Here two individuals were excluded from the sample since their estimation were detected as outliers. The regression analyses of the neuropsychological tests on the time-on-space interference were not significant for either the attention (*F*(1, 75) = 3.16, *p* = 0.08) or the working memory (*F*(1, 79) = 2.47, *p* = 0.12). Nonetheless, the overall model with short-term memory was statistically significant *F*(2, 81) = 16.66, *p* < 0.001, *η*^2^ = 0.18. Short-term memory emerged as a significant predictor of the time-on-space interference (*β* = − 0.42, *p* < 0.001), indicating that higher short-term memory capacity reduce such interference.

### Post-hoc analysis

Finally, in a mere attempt to determine whether individuals who are influenced by distance in their time estimations are also the ones affected by time in their distance estimations, we conducted a correlation analysis between spatial drift and temporal drift. The moderate correlation suggests that it is indeed the same individuals who are affected by both time and space (*r* = 0.43, *p* < 0.001).

## Discussion

The aim of the current study was to investigate the development of the interactions between time and space perception and their underlying cognitive mechanisms. To do so, children aged between 5 and 7 years participated in time estimation and distance estimation tasks. Both tasks involved the sequential presentation of three successive circles, with systematic manipulations of the interval (700, 1100 and 1500 ms) and spacing (200, 300, 400 pixels) between neighboring circles.

Our results showed that children as young as 5 years old are prone spatial biases in their time reproductions and temporal biases in their distance reproductions. These results corroborate findings from earlier developmental studies that, despite using different methods, have also observed a similar space–time interference starting at the age of 5^25,49^. Similarly, the age effect suggested a gradual development of both temporal and spatial abilities, which is consistent with many contemporary studies that have demonstrated that estimates of both space^50,51^ and time^52–54^ become more refined with age.

Contrary to our expectations, the results of our study show anti-*kappa* and anti-*tau* effects. Specifically, increasing the distances between the neighboring stimuli resulted in underestimation of durations (i.e., anti-*tau*), while increasing the durations led to underestimation of distances (i.e., anti-*kappa*). It is worth noting that this is not the first time that research has found these antagonistic effects^55–59^. For instance, Guay and Grondin^55^ observed a reversed *tau* and *kappa* effect in their study by employing a single-interval stimulus during a temporal estimation task (see Roy and colleagues^60^ for the replication of the aforementioned effect with auditory stimuli). One possibility is that certain methodologies may induce an individual processing of the stimuli comprising the sequence, rather than treating them as a cohesive whole. For instance, this could be driven by the task instructions that emphasize the temporal and spatial demarcation of the event coupled with the cognitive capacity limits or simply by the stimulus properties (e.g., number of pixels between two locations) coupled with lack of gaze fixation. Focusing on individual elements might come at the expense of not attributing motion to two successive presentations of stimuli (e.g., violating objecthood) and thereby differential processing of the spatial dimension (e.g., making it more vulnerable to cognitive resource limitations). Nonetheless, the underlying causes of these effects remain largely unexplored, and further studies are needed to draw a clear conclusion.

Regardless of being consistent with or contradicting the *tau* and *kappa* effects, the results of this study revealed interference effects of time and space. The results demonstrate that these interference effects, whether it is space-on-time or time-on-space, is consistently more pronounced in children and decreases as children grow older. Developmental researchers recently distinguish two developmental trajectories related to time processing in children^42,61^. The first—implicit processing—develops very early and results in temporal processing without awareness of time or conscious temporal reasoning. The second—explicit processing—corresponds to the progressive awareness of time and temporal reasoning between 5 and 8 years old. This explicit processing is crucial for accurate temporal estimations in tasks where individuals are aware of the need to process durations (i.e., prospective timing). According to developmental researchers on time perception, the development of explicit time judgment is closely related to cognitive development^34,35, 42, 61–63^. Thus, this study demonstrates that the interference between space and time decreases with the development of explicit temporal processing, further suggesting that the interference effects between time and space are modulated by the development of cognitive abilities.

These suppositions were supported by the series of regression analyses showing that short-term memory capacity predicts the space–time interference. Indeed, the results reveal that short-term memory significantly interacts with both space-on-time and time-on-space interferences. More specifically, children with the best short-term memory performance were less likely to be influenced by irrelevant dimensions in their productions. This result has several implications. First and foremost, the presence of a shared cognitive capacity that moderates the interference effects implies that time and space may indeed share common mechanisms, particularly in terms of mnemonic components. These findings provide support for the symmetry hypothesis. This is further supported by the post-hoc analysis, which revealed that participants who experienced interference from spatial cues in their timing performance were also the same individuals who experienced interference from temporal cues in their distance reproduction. The mutual interference between these two tasks suggests that common processes exist for the processing of temporal and non-temporal magnitudes^26^.

Furthermore, the result of short-term memory as a predictor of spatiotemporal interference provides insights into the localization of interference. Since our findings notably emphasize the existence of interference at the mnemonic stage, the interference between time and space would occur when temporal and spatial information are stored in memory. Memory interference can occur in two distinct ways. One possibility is that time and space are stored in different formats, and the different dimensions are structurally correlated, resulting in cross-dimensional interference^64^. Another possibility is that temporal and spatial information is stored in the same representational format as noisy memory representations^65,66^. In this case, noisy mental magnitudes could influence each other, leading to space–time interference^37–39^

However, it is important to note that this study does not conclusively rule out an attentional or a visuoperceptual influence and thus we cannot completely disregard the potential impact of attention during information encoding. Resultantly, our study does not allow us to rule out the existence of a specific process for estimating time and distance, or the presence of dimension-specific processes. Additionally, the significant moderation of working memory in the interference of space on time reproduction raises questions regarding the influence of executive functions. Our study also suggest that the efficiency of working memory processes may impact the occurrence of interference. Individuals with higher working memory capacity may demonstrate better cognitive control and allocation of attentional resources, enabling them to effectively process time and space, either through segregated processing or through a common processing mechanism. One may wonder why such effect was not found on the time on space interference. This may be due to the fact that time is cognitively more costly to process than non-temporal information^28^.

It is also possible that the inability of younger children to treat more than one dimension at a time due to their developmentally lower cognitive capacity might account for our findings. Under this rationale, similar interference effects would be expected between dimensions that are known to be mechanistically independent. Future studies should include control tasks to test this alternative account of our findings. Finally, we did not fully control for the possibility that children could imitate the experimenter based on the answers they provided during the demonstration (3 trials) and learning trials (3 trials).

In conclusion, this study provides compelling evidence for the existence of shared neuro-cognitive structures between time and space processing. To the best of our knowledge, this is the first behavioral study to demonstrate the presence of common cognitive components involved in both spatial and temporal processing. These findings strongly support the *symmetrical* hypothesis, suggesting a shared mechanism for the processing of time and space. This study also serves as an important catalyst for further investigation into the interactions between time and space in children. We believe that a developmental approach is crucial for gaining a deeper conceptual and empirical understanding of the overlap between time and space processing, its developmental trajectory, and underlying mechanisms.

## Data Availability

All research data are publicly available via an anonymous Open Science Framework (OSF) link. The link can be accessed by visiting [https://osf.io/neyt6/?view_only=ddf6ecfc4df44ce980663c4394cbc2f4].
